# Carbon Fiber-Reinforced PLA Composite for Fused Deposition Modeling 3D Printing

**DOI:** 10.3390/polym16152135

**Published:** 2024-07-26

**Authors:** Andong Wang, Xinting Tang, Yongxian Zeng, Lei Zou, Fan Bai, Caifeng Chen

**Affiliations:** School of Materials Science and Engineering, Jiangsu University, Zhenjiang 212013, China; wadujs@163.com (A.W.);

**Keywords:** polylactic acid, enhanced modification, mechanical properties, thermal stability, process parameters

## Abstract

Polylactic acid (PLA) composite serve as widely used filaments in fused deposition modeling (FDM) 3D printing. This study investigates the enhancement of PLA composite’s comprehensive mechanical properties and thermal stability through the incorporation of carbon fiber (CF). The influence of FDM process parameters on the mechanical properties of PLA composite is also analyzed. Results show that adding 5 wt.% CF significantly enhances the stiffness and comprehensive mechanical properties of PLA composite. The order of printing factors affecting the tensile strength of the PLA composite product is as follows: printing layer thickness, bottom plate temperature, printing speed, and nozzle temperature. Finally, optimal tensile strength is achieved under specific conditions: 0.1 mm layer thickness, 60 °C bottom plate temperature, 40 mm/s printing speed, and 215 °C nozzle temperature.

## 1. Introduction

Fused deposition modeling (FDM) is among the most widely adopted 3D printing technologies [1,2]. Commonly used printing filaments include polylactic acid (PLA) [3], nylon (PA) [4], polycarbonate (PC) [5], and acrylonitrile-butadiene-styrene (ABS) [6]. Among them, PLA can not only be completely degraded in the natural environment but also has good mechanical properties and wear resistance, which are especially suitable for FDM printing in manufacturing, medical treatment, clothing, and other industries [7]. However, PLA also exhibits significant drawbacks such as brittleness and poor heat resistance, which limit its use in applications with high-strength requirements.

To overcome these limitations, enhancing PLA through enhanced modifications to improve its comprehensive mechanical properties and thermal stability has become essential. For instance, Pavon et al. [8] studied the effects of increasing nanoparticle content on PLA modification, resulting in improved elastic modulus, tensile strength, and flexural properties. Jamnongkan et al. [9] utilized nano-ZnO to enhance PLA’s mechanical properties and antibacterial characteristics. Coppola et al. [10] enhanced PLA with layered silicate particles, demonstrating improvements in thermal stability and mechanical strength.

Fiber-reinforced PLA is also an effective improvement in terms of focusing on its high strength. Reinforcement fibers are usually available as natural or synthetic fibers. Natural fibers like corn fiber, bamboo fiber, and pineapple leaf fiber have been explored, alongside synthetic fibers such as glass fiber (GF) and carbon fiber (CF) [11,12]. Researchers like Suteja et al. [13] and Wang et al. [14] have investigated enhancing PLA with natural and glass fibers, respectively, showing improvements in tensile strength and dimensional stability. Goh et al. [15] combined GF and CF to create high-strength PLA composites using molten filament manufacturing (FFF), significantly enhancing modulus and bending strength.

Although PLA modified by nanoparticles or fiber reinforcement has better mechanical properties and other advantages, with the development of 3D printing technology, optimizing PLA reinforcement and its printing parameters for FDM applications still needs further research [16,17]. The influence of printing parameters on material mechanical performance is crucial, as demonstrated by studies such as Kechagias et al. [18]. They used the FDM manufacturing process to study the influence of layer height and nozzle temperature on tensile strength, bending stress, and impact strength at breaking. Combined with variance analysis and simulation experiments, the optimum height is about 0.2 mm and the printing temperature is 215 °C for the mechanical properties of the printing product to be the best. Elmrabet [19] et al. studied the effect of filling rate on the compression and tensile properties of the specimens made by printers. When the filling rate is 100%, the best compression and tensile performance are 2.26 GPa and 54.20 MPa, respectively. Rodriguez [20] studied some 3D printing parameters for thermoplastic polyurethane products. The parameters included floor height, wall thickness, extrusion temperature, and printing speed. From the above, it can be seen that more and more researchers are emphasizing the significance of parameters such as layer height, nozzle temperature, and printing speed on mechanical properties.

In order to improve the comprehensive mechanical properties such as tensile strength, fracture toughness, and thermal stability of PLA material, it is more suitable for FDM printing as a printing filament. In this work, carbon fiber is used to enhance the mechanical properties and thermal stability of PLA. The influence of carbon fiber on the microstructure and properties of a PLA matrix is studied, and PLA composites with excellent properties are obtained. On this basis, the effects of various printing process parameters on the mechanical properties of PLA composite products were analyzed by an orthogonal test. When tensile strength is used as the evaluation criterion, the optimal FDM printing process suitable for the CF-reinforced PLA composite is obtained.

## 2. Materials and Methods

**Figure 1:**
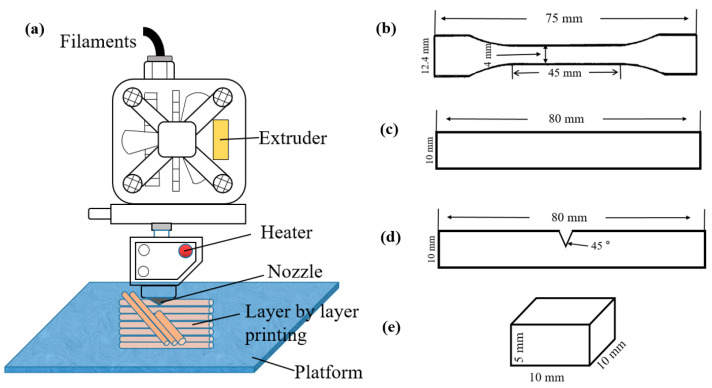
Samples printed for mechanical property testing. (**a**) Schematic diagram of the FDM molding process. (**b**) Tensile sample; (**c**) bending sample; (**d**) impact sample; (**e**) hardness sample.

PLA polymer particles (LX 175; Zhenjiang San New Material Co., Ltd., Zhenjiang, China) and CF with an average diameter of 5 µm and length of about 80 µm (Carene Technology Co., Ltd., Shenzhen, China) were dried at 60 °C for 6 h. The dried PLA and CF were fed into a twin-screw extruder (SHJ; Nanjing Jieya Basic Equipment Co., Ltd., Nanjing, China) at a ratio of 100 wt.% PLA, 95 wt.% PLA and 5 wt.% CF, and 90 wt.% PLA and 10 wt.% CF, respectively. The melt temperature of the extruder was set to 180 °C, and the extruder operated at 5.6 rpm. After the extrusion process, the pellets obtained by crushing were further dried in an oven at 60 °C for 12 h. Subsequently, a filament with a diameter of 1.75 mm was produced by extruding the pellets using a micro single-screw extruder (WSJ-12; Shanghai Xinshuo Precision Machinery Co., Ltd., Shanghai, China) at an extruder temperature of 180 °C. Finally, 3D-printed samples were fabricated by layer-by-layer deposition of the filament using a 3D printer (Raise 3D E2; Shanghai Fusion Tech Co., Ltd., Shanghai, China), as shown in Figure 1a.

The dimensions of the printed samples for mechanical testing are shown in Figure 1b–d [21,22,23,24]. The dumbbell tensile sample with a thickness of 2 mm is shown in Figure 1b. The tensile speed of the PLA sample is 5 mm/min, and the standard distance is 45 mm. The tensile strength (σ) and fracture elongation (ε) of the sample are obtained according to Equations (1) and (2).

(1) σ=F/S

(2) $$\varepsilon =\left(L-L_{0}/L_{0}\right)\cdot 100\%$$

where F is the maximum load of the sample in N, and S is the cross-sectional area in mm^2^. L is the maximum stroke of the sample, and L_0_ is the original length of the sample in mm.

Figure 1c shows the bending sample with a 4 mm thickness. The test speed was set at 5 mm/min, and the maximum pressure applied to the sample was measured. Thus, the bending strength (σ_f_) of the sample can be calculated by Equation (3).

(3) $$\sigma_{f}=\frac{3FL}{2bh^{2}}$$

where h is the sample thickness, L indicates the span length (L = 16·h, in mm), b is the sample width, and F is the maximum pressure measured.

The impact sample was printed with 45° gaps and a 4 mm thickness, as shown in Figure 1d. The impact toughness of the specimen was measured by using the XJUD-5.5 cantilever impact tester. The dimensions of the hardness sample are shown in Figure 1e. The Shore hardness tester (LX-D-2, Jiangdu Zhenbangshiyan Machinery Factory, Yangzhou City, China) was used to measure the hardness.

The microscopic morphology and structure of the broken section of the filament and printed samples were observed with a scanning electron microscope (SEM, Nova Nano SEM 450, FEI, Thermo Fisher Scientific, Waltham, MA, USA) and X-ray diffractometer (XRD, D/max 2500 PC, Rigaku, Tokyo, Japan). In addition, the diameter of the filament (d) was detected by a digital video caliper and measured three times at different positions. Three sections of filament with the same diameter were taken, with each section length L = 10 mm. The average actual density of the filament (P) is calculated according to Equation (4).

(4) $$P=\frac{4m}{\pi d^{2}L}$$

where m is the mass of filament in g, d is the diameter in mm, and L is the length of filament in mm.

## 3. Results and Discussion

### 3.1. Microstructure Analysis of the CF/PLA Composite

**Figure 2:**
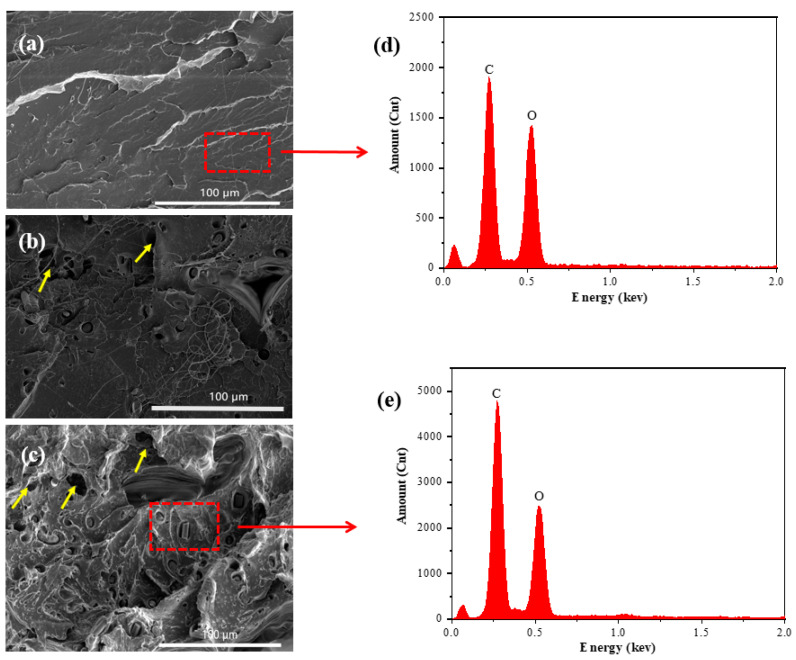
Cross-section morphology and EDS spectra of PLA composite filaments. (**a**) Pure PLA; (**b**) PLA composite with 5 wt.% CF; (**c**) PLA composite with 10 wt.% CF; (**d**) EDS spectra of pure PLA; (**e**) EDS spectra of PLA composite with 10 wt.% CF.

The cross section and surface morphology of pure PLA filament are very smooth, as shown in Figure 2a. However, when CF is added to the PLA matrix, the cross-section morphology becomes rough, as shown in Figure 2b,c. Figure 2b shows the cross-section morphology of PLA composite filament containing 5 wt.% CF. As can be seen from Figure 2b, the carbon fiber is inserted into the PLA matrix and randomly dispersed and evenly distributed. It is conducive to effective energy conduction during force and prevents stress concentration. When the amount of CF added is 10 wt.%, as shown in Figure 2c, indicated by the yellow arrow, there are obvious drawing and void phenomena on the surface, local fiber aggregation, and the section has an obvious shear deformation band formed by screw engagement and extrusion. These defects and the local concentration of fiber are not conducive to improving the comprehensive mechanical properties of composites.

Figure 2d,e shows the energy spectrum analysis of pure PLA and CF/PLA composite filaments. It can be clearly seen from the figure that pure PLA and the PLA composite only contain C and O elements, but the element content of C in the PLA composite increases sharply, and C is consistent with the main components of carbon fiber. Thus, it can be concluded that only the PLA matrix and the CF reinforcement phase are contained in the composite.

**Figure 3:**
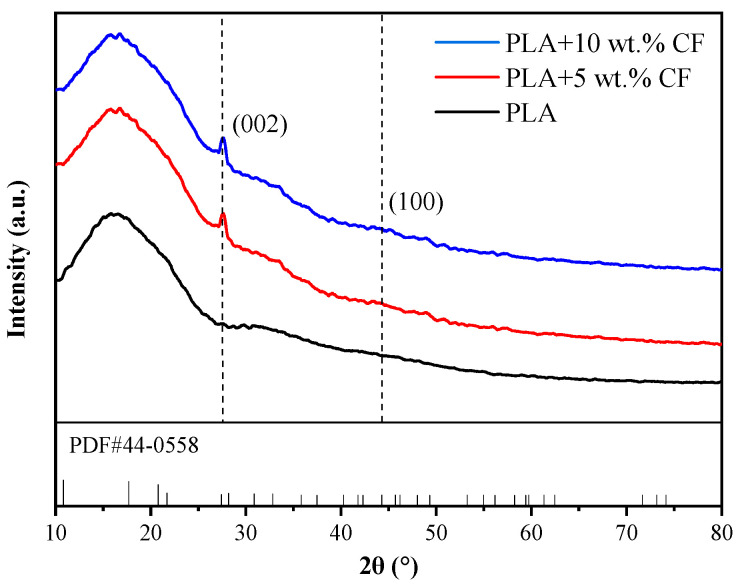
XRD diffraction patterns of PLA filaments.

The XRD diffraction patterns of the PLA filament are shown in Figure 3. In addition to the large flat peak characteristic of PLA, the characteristic peak corresponding to the CF (002) crystal plane appears near 2θ = 27°, and the weak diffraction peak corresponding to the CF (100) crystal plane appears near 2θ = 44°, which is consistent with the standard XRD diffraction pattern of CF (PDF#44-0558). It is further confirmation that CF has been successfully integrated into the PLA matrix.

### 3.2. Mechanical Properties of the CF/PLA Composite

CF embedded within the matrix and randomly distributed provides a robust skeleton structure, enhancing the density and surface hardness of the composite. It is well known that the theoretical density of carbon fiber is 1.75 g/cm^3^, and the theoretical density of pure PLA is 1.24 g/cm^3^. When the proportion of CF in the PLA matrix is certain, the theoretical density of the composite can be calculated according to the proportion relationship. Therefore, when 5 wt.% and 10 wt.% carbon fibers were added to the pure PLA matrix, the theoretical density of the composite was 1.2655 g/cm^3^ and 1.2910 g/cm^3^, respectively, as shown in Table 1.

The actual density values of the two PLA composite wires are 1.2600 g/cm^3^ and 1.2800 g/cm^3^, respectively, as shown in Table 1. Table 1 shows that with the increase in CF content, the density of the PLA composite increases. However, the theoretical density of PLA composite with 5 wt.% CF is 1.2655 g/cm^3^, while the actual density measures 1.26 g/cm^3^, reflecting a difference of 0.435%. However, for the PLA composite with 10 wt.% CF, the theoretical and actual densities differ by 0.852%. This suggests that the density of the PLA composite decreases slightly with a higher CF content. This slight reduction is due to the fact that as the CF content increases, the skeleton structure of CF may introduce voids, and excessive carbon fiber can locally aggregate, resulting in microscopic defects or voids that affect the density.

Table 1 also illustrates that the Shore hardness values of PLA composites increase with higher carbon fiber (CF) content in the PLA matrix, rising from the original 77 HD to 82 HD. This hardness increase is attributed not only to the high modulus of CF within the PLA matrix but also to the dense distribution of CF. This trend aligns with the observation that CF content in the matrix influences density.

**Figure 4:**
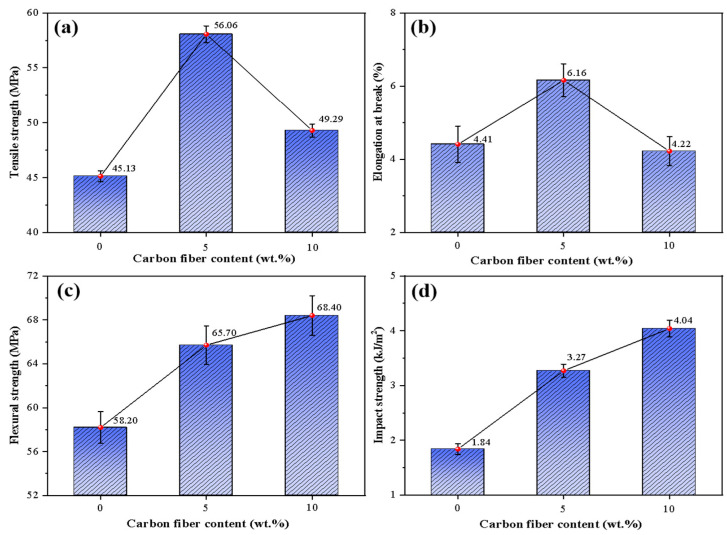
Mechanical properties of the PLA composite: (**a**) tensile strength; (**b**) fracture elongation; (**c**) bending strength; and (**d**) impact strength.

The CF dispersed in the PLA matrix plays a crucial role in enhancing the mechanical properties of the composite. When the composite is affected by an external force, the CF in the matrix will show different states. Some of the fibers are pulled out, forming voids and defects, and most of the fibers are embedded in the matrix. These CFs are pulled out or broken to absorb energy, prevent further defects, and improve the composite’s mechanical properties. The results of the tensile strength, fracture elongation, bending strength, and impact strength of the CF/PLA composite are shown in Figure 4.

As can be seen from Figure 4, the comprehensive mechanical properties of PLA composite with 5 wt.% CF are significantly improved compared with pure PLA, and the tensile strength is increased from 45.13 MPa to 56.06 MPa, with an increase of 24.2%. The elongation at break increased from 4.41% to 6.16%, the flexure strength increased from 58.20 MPa to 65.70 MPa, and the impact strength increased from 1.84 kJ/m^2^ to 3.27 kJ/m^2^. The main reason for the improvement of comprehensive mechanical properties is that carbon fiber is embedded in the PLA matrix to form a skeleton structure, and CF itself has extremely high strength and modulus [25].

When the CF content is 10 wt.%, the bending strength of the material increases from 65.7 MPa to 68.4 MPa, and the impact strength of the PLA composite containing 5 wt.% CF is also improved. However, the tensile strength of the composite decreases from 56.06 MPa to 49.29 MPa, accompanied by reduced fracture elongation. This decrease may result from an uneven distribution and agglomeration of excessive CF within the matrix, leading to diminished mechanical properties.

### 3.3. Thermal Properties of CF/PLA Composite

**Figure 5:**
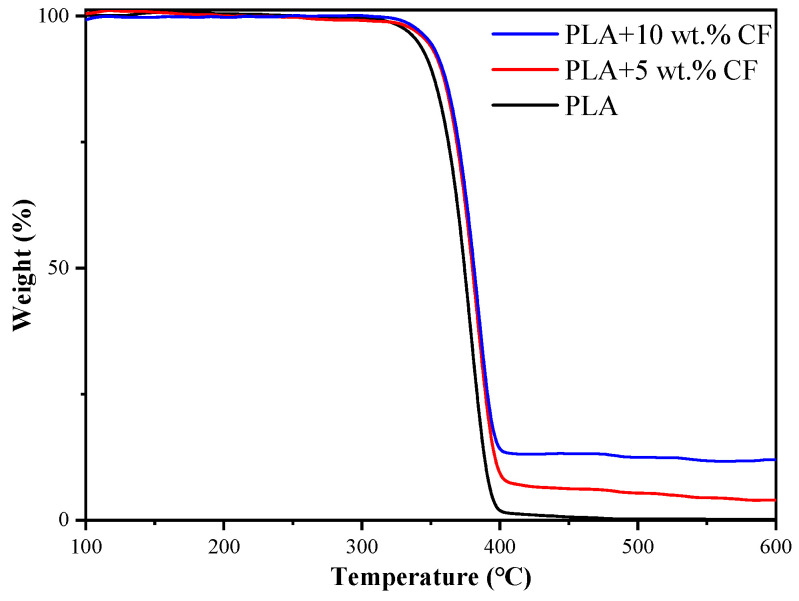
Thermal–weight curve of the PLA wire.

To explore the effect of CF on the thermal stability of PLA, the thermogravimetric (TG) curve of the PLA composite was performed, and the results are shown in Figure 5.

Figure 5 shows that the TG curve of PLA is divided into three stages: the thermal stability stage, the thermal decomposition stage, and the constant weight stage. PLA begins decomposing at 245 °C and completes decomposition at 400 °C. The TG curve of the PLA composite exhibits a similar trend to pure PLA [26], indicating that the addition of CF does not alter the decomposition process of PLA.

However, the introduction of CF enhances the thermal stability of the PLA composite. Specifically, PLA composite containing 5 wt.% CF shows an increased initial decomposition temperature to 255 °C, while PLA with 10 wt.% CF further raises this temperature to 265 °C. This improvement is attributed to the favorable thermal properties of CF, including its high thermal resistance, thermal conductivity, and low thermal expansion coefficient. These properties enable CF to effectively conduct heat and impede the mobility of PLA molecular chains when uniformly dispersed within the PLA matrix.

In conclusion, CF-enhanced PLA composites exhibit higher decomposition temperatures while retaining the inherent decomposition characteristics of pure PLA. This enhancement is attributed to CF’s beneficial effects in improving the thermal degradation resistance of PLA composite, thereby increasing the thermal stability of the composites.

### 3.4. Process Optimization for FDM 3D Printing

In FDM printing, filament is continuously fed and extruded from the nozzle after being melted at high temperatures, depositing layers to form the printed product [27]. During this process, incorrect settings, such as printing speed and nozzle temperature, can lead to filament irregularities and nozzle blockages. Additionally, the thickness of each printing layer and the temperature of the hot bed affect filament melting and internal stress within the final print, thereby influencing its quality and mechanical properties.

Therefore, optimizing parameters such as printing speed, nozzle temperature, printing layer thickness, and hot bed temperature is essential for achieving high-quality prints. PLA samples were prepared using an orthogonal test design method, which involves varying these factors across different levels to determine their optimal combinations, as detailed in Table 2.

The orthogonal test results of the PLA composite with 5 wt.% CF are shown in Table 3. As can be seen from the results, the average tensile strength of the sample with test group 3 was the highest, reaching 56.186 MPa, and the average tensile strength of the sample with test group 15 was the lowest, only 43.432 MPa.

Table 4 presents the results of the extreme difference analysis for PLA composite, where K represents the sum of indices for each factor level, k is the average of K, and R indicates the extreme difference of k, reflecting the relative importance of each factor.

According to Table 4, the factors that most significantly affect the tensile strength of PLA composite filament are ranked as follows: printing layer thickness > hot bed temperature > printing speed > nozzle temperature. This ranking suggests that printing layer thickness has the greatest impact on tensile strength, followed by bed temperature, printing speed, and nozzle temperature. The optimal printing process for achieving maximum tensile strength is identified as A1, B3, C4, and D2. This corresponds to specific parameter conditions: 0.1 mm layer thickness, 60 °C hot bed temperature, 40 mm/s printing speed, and 215 °C nozzle temperature. Under these conditions, the PLA composite filament is expected to exhibit its highest tensile strength, as determined through the extreme difference analysis method outlined in Table 4. Adjusting these parameters according to the indicated levels ensures the best balance of factors critical to achieving superior print quality and mechanical performance.

**Figure 6:**
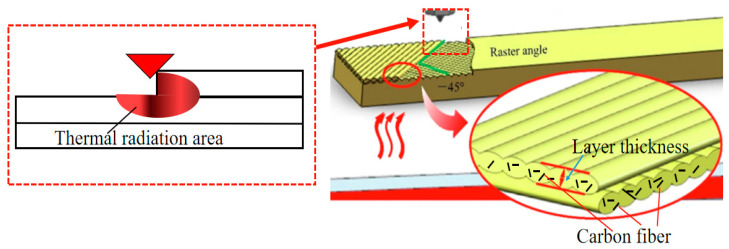
Impact of printing layer thickness and carbon fiber on composite filaments.

The impact of printing layer thickness on the tensile strength of the product primarily stems from effects related to filament re-melting and heat conduction between printed layers. For CF/PLA composite filament, which possesses high heat resistance and thermal stability, it is less susceptible to heat radiation and conduction. This characteristic is advantageous for addressing printing defects by enhancing interlayer bonding. In Figure 6, it is illustrated that when the filament is extruded and cross-printed at a 45° position, the thermal radiation area exposed to the high-temperature nozzle is minimized [28]. This setup allows for tighter packing of layers after re-melting with a smaller printing layer thickness. Consequently, this process aids in repairing interlayer defects caused during printing, thereby improving the interlayer bonding strength.

Therefore, when using a smaller printing layer thickness, the tensile strength of the sample tends to be higher due to the effective repair of interlayer defects and improved bonding. Conversely, larger printing layer thicknesses can lead to reduced strength as the bonding between layers may be less effective under these conditions.

The effect of hot bed temperature on the CF/PLA composite mainly comes from the internal stress generated by the rapid cooling of the refused filament on the bottom of the hot bed plate. Lower temperatures can result in significant internal stresses, which can adversely affect the dimensional stability of the printed object. Therefore, maintaining an appropriate hot bed temperature is crucial for minimizing shrinkage and internal stresses, thereby promoting better adhesion between printed layers.

In addition to the hot bed temperature, adjusting printing speed plays a crucial role in ensuring that the melted filament has adequate time to cool and solidify evenly. This helps in reducing the risk of stress concentration within the print.

While nozzle temperature is important for effectively melting the filament, its influence on the quality of final printed products is considered less significant compared to hot bed temperature and printing speed. Nonetheless, nozzle temperature affects interlayer adhesion and should be carefully adjusted to prevent issues such as uneven melting or excessive filament burning.

## 4. Conclusions

(1) The incorporation of carbon fiber (CF) into PLA forms a composite that benefits from a well-dispersed CF skeleton structure. This structure enhances the surface density and overall mechanical properties of the PLA composite by effectively conducting and absorbing energy. However, excessive CF content can lead to microscopic defects and aggregation.
(2) With 5 wt.% CF, the tensile strength, fracture elongation, bending strength, and impact strength of the PLA composite are measured at 56.06 MPa, 6.16%, 65.70 MPa, and 3.27 kJ/m^2^, respectively. These properties declined with higher CF content (10 wt.%).
(3) CFs with good thermal conductivity and heat resistance contribute to hindering the fracture of PLA molecular chains and enhancing thermal stability. For instance, the thermal decomposition temperature of PLA increased from 245 °C to 255 °C and 265 °C with 5 wt.% and 10 wt.% CF, respectively.
(4) Orthogonal experiments identified key factors influencing the tensile strength of CF/PLA composites as printing layer thickness, hot bed temperature, printing speed, and nozzle temperature. Achieving strong interlayer adhesion in CF/PLA prints requires using a thin printing layer thickness. Optimal hot bed temperatures and controlled printing speeds are essential to minimize internal stresses, thus maintaining the excellent mechanical strength of the printed product.
(5) To maximize tensile strength in PLA samples, optimal conditions include a printing layer thickness of 0.1 mm, a hot bed temperature of 60 °C, a printing speed of 40 mm/s, and a nozzle temperature of 215 °C. These parameters collectively contribute to producing high-quality prints with superior mechanical properties suitable for diverse industrial and functional uses.

## Figures and Tables

**Table 1:** Physical properties of PLA composites.

PLA Sample	Theoretical Density (g/cm^3^)	Actual Density (g/cm^3^)	Shore Hardness (HD)
Pure PLA	1.2400	1.2400	77
PLA + 5 wt.% CF	1.2655	1.2600	81
PLA + 10 wt.% CF	1.2910	1.2800	82

**Table 2:** The factor and level of the FDM 3D printing process.

	Factor	A (Printing Layer Thickness/mm)	B (Nozzle Temperature/°C)	C (Hot Bed Temperature/°C)	D (Printing Speed/mm/s)
Level					
1		0.10	205	30	20
2		0.15	210	40	40
3		0.20	215	50	60
4		0.25	220	60	80

**Table 3:** Orthogonal test results of the PLA composite.

Test Group	A	B	C	D	Tensile Strength (MPa)
1	1 (0.10)	1 (205)	1 (30)	1 (20)	53.792
2	1 (0.10)	2 (210)	2 (40)	2 (40)	55.822
3	1 (0.10)	3 (215)	3 (50)	3 (60)	56.186
4	1 (0.10)	4 (220)	4 (60)	4 (80)	55.220
5	2 (0.15)	1 (205)	2 (40)	3 (60)	50.544
6	2 (0.15)	2 (210)	1 (30)	4 (80)	47.646
7	2 (0.15)	3 (215)	4 (60)	1 (20)	54.758
8	2 (0.15)	4 (220)	3 (50)	2 (40)	53.134
9	3 (0.20)	1 (205)	3 (50)	4 (80)	47.772
10	3 (0.20)	2 (210)	4 (60)	3 (60)	49.508
11	3 (0.20)	3 (215)	1 (30)	2 (40)	47.982
12	3 (0.20)	4 (220)	2 (40)	1 (20)	48.514
13	4 (0.25)	1 (205)	4 (60)	2 (40)	49.508
14	4 (0.25)	2 (210)	3 (50)	1 (20)	48.094
15	4 (0.25)	3 (215)	2 (40)	4 (80)	43.432
16	4 (0.25)	4 (220)	1 (30)	3 (60)	44.174

**Table 4:** Range analysis of the CF/PLA composites.

Range	A	B	C	D
K1	221.02	201.62	193.59	205.16
K2	206.08	201.07	198.31	206.45
K3	193.78	202.36	205.19	200.41
K4	185.21	201.04	208.99	194.07
k1	55.26	50.40	48.40	51.29
k2	51.52	50.27	49.58	51.61
k3	48.44	50.59	51.30	50.10
k4	46.30	50.26	52.25	48.52
R	8.95	0.33	3.85	3.09

## Data Availability

The authors confirm that the data supporting the funding of this study are available within the article.
